# The effect of masks on the recognition of facial expressions: A true-to-life study on the perception of basic emotions

**DOI:** 10.3389/fpsyg.2022.933438

**Published:** 2022-12-22

**Authors:** Michael Christian Leitner, Verena Meurer, Florian Hutzler, Sarah Schuster, Stefan Hawelka

**Affiliations:** ^1^Salzburg University of Applied Sciences, Salzburg, Austria; ^2^Centre for Cognitive Neuroscience (CCNS), University of Salzburg, Salzburg, Austria; ^3^Department of Psychology, University of Salzburg, Salzburg, Austria

**Keywords:** emotion perception, face masks, social interaction, interpersonal communication, video stimulus, basic emotions, COVID-19

## Abstract

Mouth-to-nose face masks became ubiquitous due to the COVID-19 pandemic. This ignited studies on the perception of emotions in masked faces. Most of these studies presented still images of an emotional face with a face mask digitally superimposed upon the nose-mouth region. A common finding of these studies is that smiles become less perceivable. The present study investigated the recognition of basic emotions in video sequences of faces. We replicated much of the evidence gathered from presenting still images with digitally superimposed masks. We also unearthed fundamental differences in comparison to existing studies with regard to the perception of smile which is less impeded than previous studies implied.

## 1. Introduction

In the light of the COVID-19 pandemic, face masks are used in everyday life to reduce the transmission of the SARS-CoV-2 virus. However, face masks do not only play a central role in infection control, but they also have an impact on social interaction. For the first time in (western) history, the faces of communication partners have been systematically obscured in public for months and years. According to Carbon (2020), about 60–70% of facial areas relevant for the expression of emotions are thus hidden. Since the end of the pandemic cannot yet be foreseen, one must assume that face masks will accompany us for some time to come. From a psychological perspective the question arises to what extent these masks influence the recognition of facial emotion for interlocutors. The present study is the first of its kind to use naturally moving faces to investigate experimentally how face masks affect emotion recognition.

The most popular methodology for categorizing facial emotions is the so-called *Facial Action Coding System* (FACS) by Ekman et al. (2002). FACS is a categorical system for determining facial expressions based on the smallest visually perceptible facial movements, called *Action Units* (AUs). Psychometric evaluations of the FACS show good to excellent interrater reliability in coding the occurrence, intensity, and timing of specific AUs (Sayette et al., 2001). Likewise, several studies demonstrated high validity when comparing the FACS manual with computer-based methods for analyzing facial expressions and thus various automated detection systems are continuously developed and further improved (e.g., Bartlett et al., 1999; Cohn et al., 1999; Pantic and Patras, 2006; Baltrusaitis et al., 2018; Yudiarso et al., 2020). Most of these computers assisted and automated systems are based on the so-called “Basic emotions” paradigm - that is; Sadness, Anger, Surprise, Fear, Disgust, Contempt and Happiness - also formulated by Ekman (1999) and widely accepted in the scientific community as valid constructs of interculturally observable human behavior.

Since the start of the pandemic, several studies have investigated the effects of facial masks on emotion recognition and interpretation. However, so far only static pictures of displayed emotions (obtained from, e.g., MPI Facial Expression Database (Kaulard et al., 2012), Matsumoto and Ekman database (1988) or DANVA2-AF Diagnostic Analysis of nonverbal Accuracy) have been widely used in the respective study designs. As these databases offer only maskless faces, masks have been simply added digitally and compared to the original faces in these experimental designs (e.g., Carbon, 2020; Ruba and Pollak, 2020; Bani et al., 2021; Calbi et al., 2021; Gori et al., 2021; Grundmann et al., 2021; Marini et al., 2021; Pazhoohi et al., 2021; Sheldon et al., 2021; Grahlow et al., 2022; Kim et al., 2022). Hofmann et al., 2021 used - additionally to their also experimentally used and digitally altered still photographs of displayed emotions - a multimethod setting that provides a more holistic insight into human perception and experience with masked and unmasked frontline employees from a customer viewpoint. Kastendieck et al. (2022) digitally placed surgical masks on existing video footage. However, the predominant use of “static-image-methodology” is not surprising as even before the pandemic, empirical questions on the perception and interpretation of nonverbal facial behavior were predominantly evaluated with still images (Paiva-Silva et al., 2016).

Only the minority of studies conducted so far report no general strong influence of masks on emotion recognition (Ruba and Pollak, 2020; Calbi et al., 2021; Kastendieck et al., 2022). In contrast, most studies conclude an overall significant influence on the perception and interpretation of facial emotions when a mask is worn:

- Most studies find that recognition of *anger* is impaired when the corresponding faces were presented with a mask (Carbon, 2020; Bani et al., 2021; Grahlow et al., 2022; Pazhoohi et al., 2021; Kim et al., 2022).
- The detection of *disgust* also consistently showed significant limitations due to wearing a mask (Carbon, 2020; Grahlow et al., 2022; Pazhoohi et al., 2021; Kim et al., 2022). Additionally, the recognition of disgust was the most impaired of all emotions in two studies (Carbon, 2020; Grahlow et al., 2022).
- *Sadness* was also significantly less detectable with mask in Bani et al. (2021), Carbon (2020), Grahlow et al., (2022), Kim et al. (2022), Marini et al. (2021), and Pazhoohi et al. (2021). In contrast, Kastendieck et al. (2022) - conducting a video-based study design, but with only digitally added masks - found no difference in the expression of sadness between masked and unmasked trials.
- Except for studies from Marini et al. (2021) and Pazhoohi et al. (2021), masks did not show any limitations in detecting *fear* (Carbon, 2020; Bani et al., 2021; Grahlow et al., 2022). Kim et al. (2022) additionally showed that covering the eye region by wearing sunglasses leads to significant limitations in emotion recognition of fear - while there were no significant differences between stimuli with and without mouth-nose protection.
- Regarding *happiness*, previous studies provide the most inconsistent results: Bani et al. (2021), Carbon (2020), Kim et al. (2022), Marini et al. (2021), and Pazhoohi et al. (2021) found that joyful faces were significantly worse to identify while wearing a mask. In contrast, in the studies by Grahlow et al., (2022), Hofmann et al. (2021) and Kastendieck et al. (2022) emotion recognition with a mask was not impaired for happiness. Furthermore, Sheldon et al. (2021) were the first and so far only study to investigate the effect of mouth-nose protection on Duchenne (sincere) vs. social (insincere) smiles. Study participants were presented with photos of faces showing either a Duchenne smile, a social smile, disgust, or a neutral expression with and without a mask. Afterwards, the subjects were asked to rate to what extent the individual photos depicted the four emotions. Results showed that a masked social smile was perceived as significantly more neutral and less friendly than an unmasked social smile. In case of the Duchenne smile, in contrast, the mask affected the perception of friendliness significantly less.
- In most studies, a masked *neutral* expression could still be identified as such (Carbon, 2020; Grahlow et al., 2022; Marini et al., 2021; Kim et al., 2022); only in the study by Pazhoohi et al. (2021) participants had more difficulty to identify neutral faces when they were masked.

In brief, the key findings of the conducted studies to date can be summed up as follows.

- Anger, disgust, sadness, happiness (social as well as Duchenne) are significantly harder to identify when masked
- The identification of fear and neutral expressions is not affected by masks

As outlined above, most studies that found a significant influence of masks on the ability to perceive emotions have been conducted with photos - with masks digitally superimposed. These depictions are static, mostly showing the emotional expression at its “peak” without the variations an emotional expression encompasses in its due course. For a more true-to-life evaluation of the perception of emotion in masked faces, video material is - most probably - more suitable. Facial emotions are composed of a multitude of simultaneously (more or less intensively) activated muscle groups. Moreover, these signals are transient - affecting the communication partner over a period of time in different degrees. A static image cannot adequately reflect this complexity. In addition, a massively limiting factor in the perception of emotions in static pictures may be that the mask does not move, nor slip or wrinkle in accordance with the facial movements of expressing the emotion.

The objective of the present study was to re-investigate the perception of emotions in masked faces with an ecologically valid procedure, that is, with video sequences of facial expressions of emotions. We expect little differences to previous studies for rather “static” emotional expressions such as sadness which only involves subtle movements of facial muscles. With regard to the expression of happiness - particularly a “dishonest” (i.e., social) smile - participants may detect this expression due to the movement (i.e., elevation) of the face mask. This finding would be discrepant to existing studies with still images which reported that masked smiles were perceived as a neutral expression. An honest smile might be the easiest emotion to perceive because of the elevation of the mask and the presence of the Duchenne marker.

## 2. Materials and methods

### 2.1. Participants

A total of 267 participants (188 female, 78 male and 1 divers) with a mean age of 31 years (SD = 15) participated in the online survey. About half (57%) of the sample were students, the other participants had various professions. The students were reimbursed for their efforts with course credits. Every participant additionally had the chance to win vouchers from an online marketplace. Mean completion time for the whole experiment was 32 min (SD = 14).

### 2.2. Material

**Figure 1:**
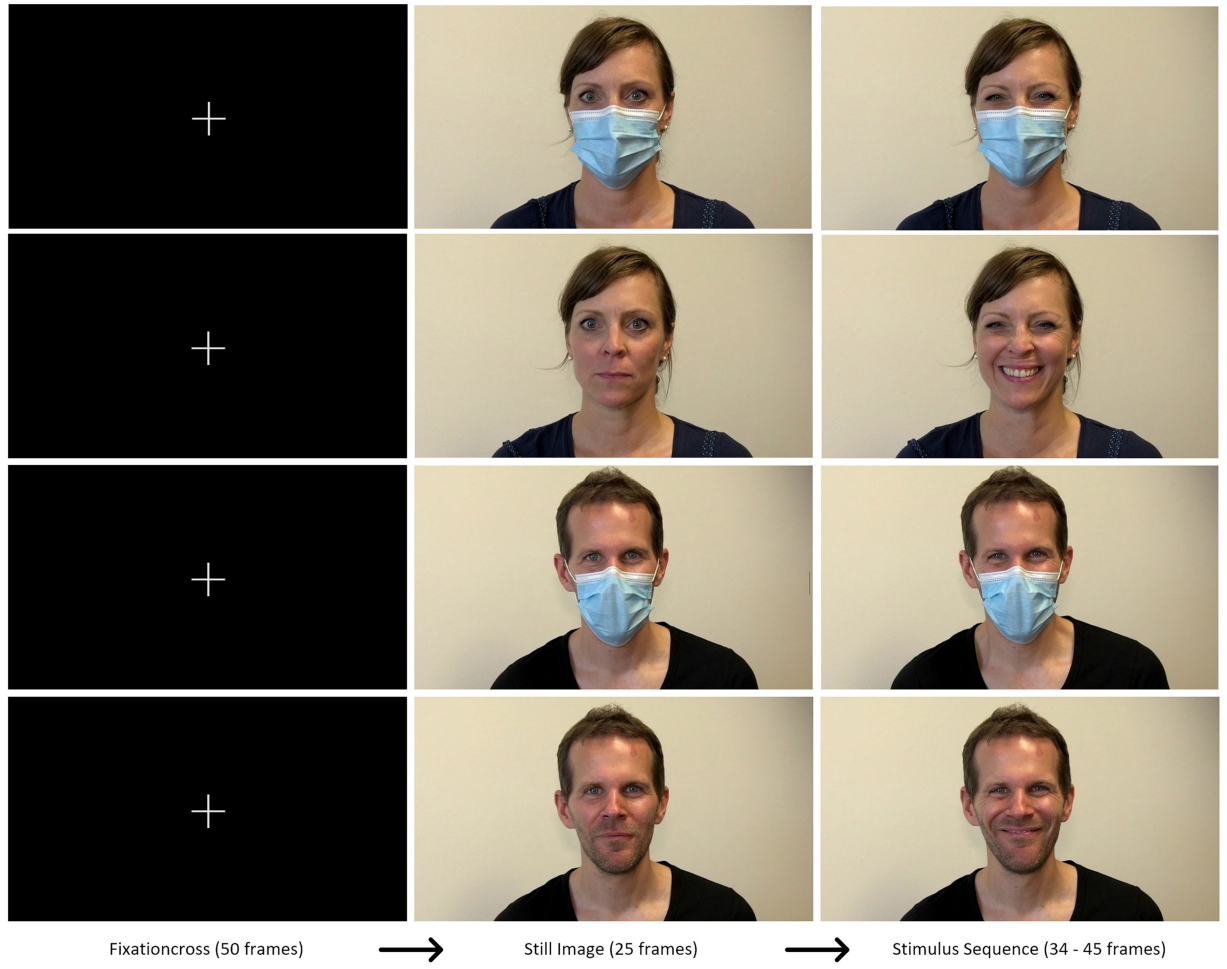
Schematic illustration of experimental trials displaying the Duchenne condition from both actors with and without mask.

The video clips depicted a caucasian actor and an actress performing the action units (AUs), that is, happiness (social vs. Duchenne smile), anger, disgust, sadness, fear and neutral. Written informed consent was obtained from the actors for publication of images and video material. The actors - with years of professional experience - were thoroughly instructed on the relevant features in facial expression that defined the respective AUs. If necessary, the actors were re-instructed and given feedback during the preparation and recording of the video clips based on the FACS manual (Ekman et al., 2002). The AUs were repeated with the face mask (conventional surgical mask; see Figure 1). We paid attention that the (observable) facial expressions with and without the face masks were as similar as possible both during preparation, as well as during the video post-production and selections of the numerous final video clips. A pre-test assured that the depicted emotions were correctly identified when no mask was worn. Furthermore, we validated our material with the OpenFace toolkit (Baltrusaitis et al., 2018) on facial action recognition *via* several parameters such as facial landmark detection. The results of this analysis are presented in the Supplementary Material and coincide largely with AU definitions based on the FACS manual.

### 2.3. Procedure

The experiment was conducted online *via* the LimeSurvey plattform (Limesurvey GmbH, 2012). Access was granted between January 15 and March 29, 2022 - a period of time in which mask wearing was mandatory in all publicly accessible places across central Europe, such as supermarkets, public transport, educational institutions, asf.

The procedure of the present experiment is illustrated in Figure 1. A fixation cross on black background preceded each trial (2 s). Thereafter, a video clip presenting a basic emotion, that is, happiness (social vs. Duchenne smile), anger, disgust, sadness, fear, or a neutral expression - either masked or unmasked - was presented in such a way that the tip of the nose of the respective actor/actress aligned to the center of the screen. After a brief still image of 1 s (25 frames), the respective emotion was performed between 34 and 45 frames (about 2 s). Trial presentation was randomized for every participant. Each portrayed emotion was presented once to participants in four different versions; that is: male masked, male unmasked, female masked, female unmasked. Two familiarization trials (neutral expression) preceded the experimental run. After each video clip, the participants were asked to identify the displayed emotion, rate its intensity and rate their confidence in emotion identification on a 7-point likert scale. For the emotion happiness (i.e., social smile and Duchenne smile), the participant was further asked to rate how honest they felt the portrayed emotion.

### 2.4. Statistical analysis

Chi-Square tests were performed for the investigation of rating regarding participants’ perception of displayed facial emotion and Wilcoxon signed-rank test was used for the investigation of participants’ certainty, and in case of the happiness condition also the honesty of the displayed, masked, and unmasked facial emotion. We corrected (Bonferroni) the resulting *p*-values for the multiple comparisons. Furthermore, we report effect sizes, that is, Cramer’s V for the perception of the emotions and Cohen’s d for the (Likert-scaled) measures of certainty, intensity honesty.

## 3. Results

### 3.1. Perception

Table 1 presents the mean percentage of recognizing the displayed emotion without and with facial mask and the corresponding results from the Chi-square comparison. In general, the recognition of smiles (both Duchenne and social smiles) and neutral faces were high with means of above 90%. Anger and sadness were the least often correctly identified emotions. The Chi-square tests revealed that Duchenne smiles, anger, disgust, and sadness were statistically significantly harder to recognize in masked than in unmasked faces. In contrast, social smiles, fear, and neutral expressions show no statistically significant difference in recognition with and without mask.

**Table 1:** Mean percentage of recognizing the displayed emotion without and with a facial mask, the result from the Chi-square comparison and effect sizes (Cohen’s d).

Emotion	No mask	Mask	*X* ^2^	*p*-corr	Cramer’s V
Duchenne	96	88	11.15	<0.01	0.20
Social smile	93	91	0.40	1.00	0.04
Anger	73	42	51.52	<0.001	0.44
Fear	86	85	0.14	1.00	0.02
Sadness	60	27	56.36	<0.001	0.46
Disgust	80	48	57.38	<0.001	0.46
Neutral	94	95	0.31	1.00	0.03

Sadness was more often misperceived as fear (35%) than correctly perceived as sadness. It was also often misperceived as disgust (26%). Disgust was often erroneously perceived as anger (24%) and fear (21%). Anger was often misperceived as disgust (29%) and less often as fear (17%). A complete confusion matrix is provided in the Supplementary material. We also provide the percentage of emotion recognition separately for the actress and the actor in the Supplement. Importantly, this separate analysis revealed the same pattern of results as the analysis of the male–female average. However, the actress elicited higher recognition rates of anger and sadness than for the male actor (whose masked sadness was utterly imperceivable with a recognition rate of only 4%). The actor, to the contrary, elicited a slightly higher recognition rate for the Duchenne smile than the actress.

### 3.2. Certainty

Table 2 presents the mean subjective certainty with which the participants recognized the displayed emotion. In general, the certainty was high for both smiles (Duchenne and social) and for neutral expressions. The participants felt less certain in response to sad, disgusted, and fearful faces. Statistically, the difference between the unmasked and the masked faces was significant for each of the emotions. Expectedly, the certainty of recognizing the emotion was higher for the unmasked faces. Numerically, the differences in certainty were highest for disgust and sadness followed by the Duchenne smile. The separate analyses for the female and the male actor revealed a similar pattern. A noteworthy difference, however, was that the certainty of perceiving anger was higher for the unmasked actress than for the unmasked actor (*Z* = 11.23, *p* < 0.001).

**Table 2:** Mean scores of the ratings how certain the participants were in recognizing the displayed emotion without and with facial mask, the result from the Wilcoxon test and effect sizes (Cohen’s d).

Emotion	No mask	Mask	*Z*	*p*-corr	d
Duchenne	6.20	5.26	8.93	<0.001	0.76
Social smile	6.03	5.31	7.97	<0.001	0.56
Anger	5.31	3.99	11.21	<0.001	1.08
Fear	5.54	5.07	5.60	<0.001	0.38
Sadness	5.14	3.85	11.13	<0.001	1.08
Disgust	5.21	3.84	11.50	<0.001	1.10
Neutral	6.08	5.44	6.66	<0.001	0.50

### 3.3. Intensity

Table 3 shows the mean score of how intense the participants perceived the displayed emotion without and with a facial mask. Expectedly, the neutral expression elicited the lowest intensity rating with little difference whether or not the face wore a mask (although the difference is statistically significant). Fear, disgust, sadness, and the Duchenne smile scored highest in the intensity ratings. For fear and the Duchenne smile it made little difference whether or not the faces were masked. Sadness, disgust and - on a lower level - social smile were perceived as more intense without a mask than with a mask. Separate analyses for the actress and the actor revealed the same pattern of results.

**Table 3:** Mean scores of the ratings how intense the participants perceived the displayed emotion without and with facial mask, the result from the Wilcoxon test and effect sizes (Cohen’s d).

Emotion	No mask	Mask	*Z*	*p*-corr	d
Duchenne	4.45	4.22	2.57	0.07	0.18
Social smile	3.55	3.06	6.27	<0.001	0.39
Anger	4.22	3.81	5.44	<0.001	0.37
Fear	4.87	4.84	0.43	1.00	0.03
Sadness	4.62	4.05	7.31	<0.001	0.56
Disgust	4.63	4.10	6.74	<0.001	0.48
Neutral	2.97	2.73	2.99	0.02	0.12

### 3.4. Honesty

**Figure 2:**
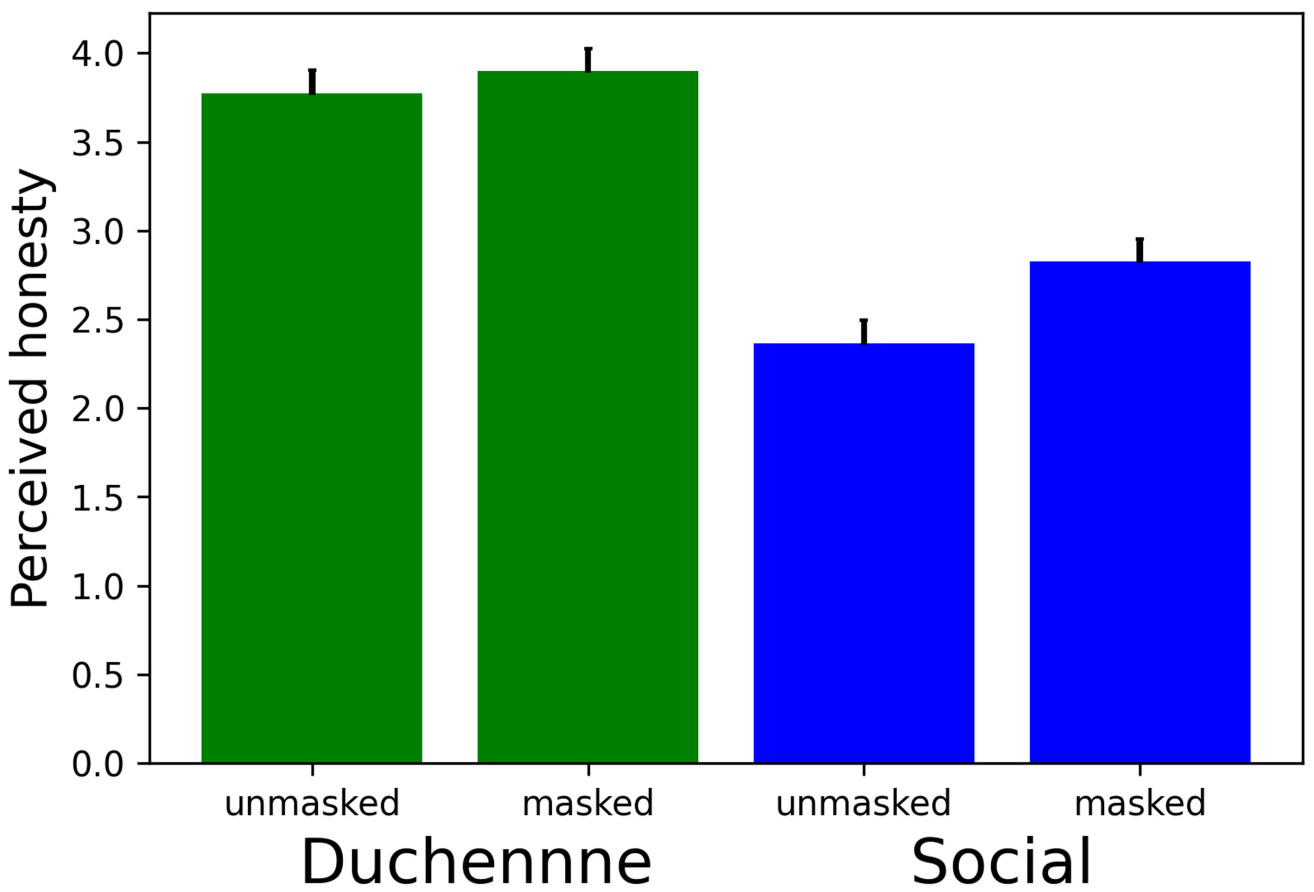
Mean honesty ratings for unmasked and masked smiles separately for an honest (Duchenne) and a dishonest (social) smile. Error bars indicate the 95% confidence interval.

We let the participants rate the honesty of the Duchenne and the social smile. The result is depicted in Figure 2. Expectedly, participants rated the Duchenne smile in both conditions (unmasked and masked; *M* = 3.77 and 3.90, respectively) significantly more honest than the social smile (*M* = 2.37 and 2.83; *Z* = 10.22 and *Z* = 11.58, *p*s < 0.001, *d*s > 1.0). Less expected, the participants perceived the social smile on average more honest in masked faces than in unmasked faces (*Z* = 5.57, *p* < 0.001, *d* = 0.37). The separate analysis revealed that this effect was present for both the actress and the actor (*Z*s > 2.30, *p*s < 0.05).

## 4. Discussion

The study set out to investigate the effect of wearing a facial mask on the perception of emotion in video sequences of faces. Considering the ubiquity of face masks due to the COVID-19 pandemic, numerous studies investigated this issue. However, these studies used portraits of faces (often from repositories) and the masks had been superimposed on the still images. We argued that the recognition of emotion in masked, but animated faces may differ from recognizing the same emotion in still images. One reason is that the expression of an emotion is a transient process and perceiving it evolving may contribute to recognition. Another and possibly an even higher weighting factor is that one can perceive the movement of the mask when the emotion unfolds as, for example, an elevation of the mask in case of a smile. Our findings are, in many aspects, similar to the findings of previous studies. There are, however, also notable differences. The results on Duchenne and social happiness seem particularly interesting. As described above, this is also where one finds the greatest differences in existing studies.

An honest smile, that is the Duchenne smile, is exceptionally easy to recognize in a fully visible face as evinced by the highest (cloze-to-ceiling) recognition rate of all the emotions of the present study. Besides the raising of the corners of the lips, this sort of smile is further characterized by the activation of the orbicularis oculi muscle and pars orbitalis muscle. Thus, the smile still has a high recognition rate even when the lower part of the face is masked (still close to 90%). However, the difference in recognition rate in the unmasked and the masked condition was statistically significant. Thus, a facial mask does impede the perception of an honest smile, albeit at a very high level of successful recognition (see also Sheldon et al., 2021).

Social (“dishonest”) smiles also had a high recognition rate and - in contrast to the Duchenne smile - the mask had no (significant) effect on recognition. This finding is interesting as social smiles are primarily communicated through facial regions located under the mask. Possibly, the elevation and wrinkling of the mask plays a role in recognizing this kind of smile. Studies with still images reported that smiles are more difficult to perceive when masked (e.g., Carbon, 2020; Bani et al., 2021; Marini et al., 2021; Pazhoohi et al., 2021; Kim et al., 2022). To illustrate, Sheldon et al. (2021) conducted a study with still images of masked faces showing Duchenne smiles and social smiles. They reported that masked social smiles became non-smiles. The discrepancy of their findings and ours emphasizes the value of studying emotion perception with videos of facial expressions.

With regard to the perceived certainty of participants’ interpretation of displayed emotions, the consistent difference between masked and unmasked conditions for all emotions (including neutral expressions) is noteworthy. The differences in the rating on the 7-point Likert scale amount to an average reduction of about one scale value (−0.96). The largest reduction we found was for disgust (−1.37), the smallest for anger (−0.47). However, the latter finding has to be put into perspective, because for anger we found a substantial difference in the certainty ratings for the female and the male actress. The participants were much more certain about perceiving anger in the face of the actress. In sum, the mask exerts an influence on the certainty of perceiving emotions. Likewise, masks also play a role - albeit smaller than for the rating of certainty - in the perceived intensity of the displayed emotions. All but one emotional expression was perceived less intensive in the masked faces. Perceiving the intensity of fear was not (significantly) affected by the mask. This finding together with similar recognition rates in unmasked and masked faces (consistent with Carbon, 2020; Bani et al., 2021; Grahlow et al., 2022) conforms to the evidence that the most influential feature for recognizing fear are widened eyes (e.g., Yarbus, 1967; Kim et al., 2022).

Moreover, our results are particularly interesting with respect to the perceived honesty of the two displayed happiness conditions, that is, the Duchenne and the social smile. We found that the perceived honesty of social smiles with a mask is different from social smiles without a mask in the unexpected direction: The participants tended to rate a “fake” smile with a mask more “genuine” than without a mask. This was the case in the averaged data and for both the actress and the actor in the separate analyses (so the next time you are selling a broken car, put on a mask). Since in the upper, freely visible areas of the face muscle groups are less activated than in an “honest” Duchenne smile - little information about the displayed emotion of a social smile is available there. Thus, our finding could represent an expectancy effect that results from the movement of the mask due to the smile underneath. Since the smile cannot be processed in its entirety due to the mask, interlocutors may tend to automatically evaluate more in the direction of honesty. This is, of course, a mere speculation, but may ignite further research into the possible source of this unexpected effect.

It may be a little excursion but comparing pre-pandemic studies with the findings and implications of the studies on the effect of wearing masks on emotion recognition may be worthwhile. To illustrate, Boucher and Ekman (1975) and Wegrzyn et al. (2017) found that the most important diagnostic information for the identification of fear is in the eye area. Thus, it makes perfect sense that subjects in the mask studies were, for the most part, able to identify anxiety (e.g., Carbon, 2020; Bani et al., 2021; Grahlow et al., 2022; Kim et al., 2022). For the identification of disgust, in contrast, pre-pandemic studies (Boucher and Ekman, 1975; Wegrzyn et al., 2017) showed a clear focus on AUs in the mouth and cheek area. Accordingly, it is interesting to observe that the mask studies also showed strong limitations in emotion recognition specifically for disgust (Carbon, 2020; Grahlow et al., 2022; Pazhoohi et al., 2021; Kim et al., 2022). This is also true for happiness, the slightly inconsistent results of the mask studies (Carbon, 2020; Bani et al., 2021; Grahlow et al., 2022; Hofmann et al., 2021; Marini et al., 2021; Pazhoohi et al., 2021; Kim et al., 2022) can possibly be explained by the fact that Boucher and Ekman (1975) observed a focus on both mouth and eye region when viewing happy faces, whereas Wegrzyn et al. (2017) observed a focus on the mouth. Barrick et al. (2021) reported that there are some indications that eye cues could become more important in the reading of emotions the longer masks are worn by the general public. Consequently, research on the perception of emotional facial expressions before the introduction of masks in the course of the COVID-19 pandemic may become less representative.

The particular challenge of the present study was the creation of the video stimulus material. We had to be meticulous that in both conditions (mask / no mask) the visible (and hidden) AUs were activated as identically as possible. For this reason, we opted for a multi-stage validation process, starting with an particularly explicit instruction of the actors, through feedback and corrections during filming and the selection of the best matches in the course of post-production, to evaluation with OpenFace (see Supplementary material). The OpenFace analysis revealed that the actress and the actor expressed the smiles - both Duchenne and social - very similar, that is, by activation of the same action units (AU). The only noteworthy difference was that the actress in the Duchenne condition smiled with parted lips (i.e., open-mouthed; AU25), whereas the actor did not. Sadness and disgust were also expressed similarly by both actors, but there were quantitative differences in the activation strength of the action units. The actors did differ in expressing anger which probably contributed to the different recognition rates in this condition and leads us directly to discussing the study’s limitations.

## 5. Limitations

It is clearly a limitation that we used stimuli from only two actors. The creation of such stimuli is costly with respect to time (see above) and human resources (if one opts, as we did, for professional actors). A replication with emotional expressions of more different faces would be time consuming, but expedient. In a similar vein, we also did not study the whole spectrum of basic emotions. Future studies may include surprise and contempt. With hindsight, another critical aspect is that we used surgical masks which - at that time - were omnipresent. In the meantime, the wearing of surgical masks waned and FFP2 masks became much more common. Had we used FFP2 masks, the findings might differ. FFP2 masks are more rigid and sit tighter on the face. Thus, one may reason that this sort of mask may affect the perception of emotional facial expressions to a greater extent and this may be particularly so for the social smile. A follow-up study with FFP2 masks would clarify this issue.

## 6. Conclusion

Video footage of facial emotions creates more informative context than still images. Especially against the background of topical questions regarding the effects of masks on interpersonal communication, digitally superimposed masks on photographs are artificial compared to actually worn masks in video material. Especially for more complex research questions - that go beyond answering the principal perception of basic emotions (such as, e.g., the distinction between honest and dishonest smiles) specially created stimulus material should play a more prominent role in future investigations.

Although some differences in emotion recognition, perceived certainty and intensity between masked and unmasked faces seem rather small in absolute terms, we can conclude that masks impede the interpretation of facial emotions and reduce perceived certainty and intensity. We also found, surprisingly, that masked social smiles were perceived as more honest than social smiles which were fully perceivable. Still, one perceives a Duchenne smile as more honest than a social smile regardless of whether the opponent’s face is fully visible or only half visible due to a mouth-nose mask. Thus, smile and mean it - during the pandemic and afterwards - it will be appreciated!

## Data availability statement

The raw data supporting the conclusions of this article will be made available by the authors, without undue reservation.

## Ethics statement

Ethical review and approval was not required for the study on human participants in accordance with the local legislation and institutional requirements. The participants provided their written informed consent to participate in this study. Written informed consent was obtained from the individual(s) for the publication of any potentially identifiable images or data included in this article.

## Author contributions

FH, ML, VM, and SH: conception and design. ML and VM: data acquisition. SH, ML, and VM: analysis and interpretation of data. ML, SH, VM, and SS: writing publication. ML, FH, SS, and SH: critical revision of publication. ML and SH: supervision. ML and FH: resources. VM and SS: technical administrative and support. All authors contributed to the article and approved the submitted version.

## Funding

The research was supported by the Austrian Science Fund (FWF; P31299).

## Conflict of interest

The authors declare that the research was conducted in the absence of any commercial or financial relationships that could be construed as a potential conflict of interest.

## Publisher’s note

All claims expressed in this article are solely those of the authors and do not necessarily represent those of their affiliated organizations, or those of the publisher, the editors and the reviewers. Any product that may be evaluated in this article, or claim that may be made by its manufacturer, is not guaranteed or endorsed by the publisher.

## References

[ref1] Baltrusaitis T. Zadeh A. Lim Y. C. Morency L. P. (2018). “Open Face 2.0: Facial Behavior Analysis Toolkit.” in *IEEE International Conference on Automatic Face & Gesture Recognition*.

[ref2] Bani M. Russo S. Ardenghi S. Rampoldi G. Wickline V. Nowicki S. Jr.. (2021). Behind the mask: emotion recognition in healthcare students. Medical Science Educator 31, 1273–1277. doi: 10.1007/s40670-021-01317-8, PMID: 34035987 PMC8136366

[ref3] Barrick E. M. Thornton M. A. Tamir D. I. (2021). Mask exposure during COVID-19 changes emotional face processing. PLoS One 16:e0258470. doi: 10.1371/journal.pone.0258470, PMID: 34637454 PMC8509869

[ref4] Bartlett M. S. Hager J. C. Ekman P. Sejnowski T. J. (1999). Measuring facial expressions by computer image analysis. Psychophysiology 36, 253–263. doi: 10.1017/S0048577299971664 10194972

[ref5] Boucher J. D. Ekman P. (1975). Facial areas and emotional information. J. Commun. 25, 21–29. doi: 10.1111/j.1460-2466.1975.tb00577.x 1127138

[ref6] Calbi M. Langiulli N. Ferroni F. Montalti M. Kolesnikov A. Gallese V.. (2021). The consequences of COVID-19 on social interactions: an online study on face covering. Sci. Rep. 11:2601. doi: 10.1038/s41598-021-81780-w, PMID: 33510195 PMC7844002

[ref7] Carbon C. (2020). Wearing face masks strongly confuses counterparts in Reading emotions. Front. Psychol. 11:566886. doi: 10.3389/fpsyg.2020.566886, PMID: 33101135 PMC7545827

[ref8] Cohn J. F. Zlochower A. J. Lien J. Kanade T. (1999). Automated face analysis by feature point tracking has high concurrent validity with manual FACS coding. Psychophysiology 36, 35–43. doi: 10.1017/S0048577299971184, PMID: 10098378

[ref9] Ekman P. (1999). “Basic emotion,” in Handbook of cognition and emotion. eds. Dalgleish T. Power M. (Hoboken: New Jersey), (John Wiley & Sons), 45–60.

[ref10] Ekman P. Friesen W. V. Hager J. C. (2002). The facial action coding system: A technique for the measurement of facial movement. San Francisco, CA: Consulting Psychologists Press.

[ref11] Gori M. Schiatti L. Amadeo M. B. (2021). Masking emotions: face masks impair how we read emotions. Front. Psychol. 12:669432. doi: 10.3389/fpsyg.2021.669432, PMID: 34113297 PMC8185341

[ref12] Grahlow M. Rupp C. I. Derntl B. (2022). The impact of face masks on emotion recognition performance and perception of threat. PLoS One 17:e0262840. doi: 10.1371/journal.pone.0262840, PMID: 35148327 PMC8836371

[ref13] Grundmann F. Epstude K. Scheibe S. (2021). Face masks reduce emotion-recognition accuracy and perceived closeness. PLoS One 16:e0249792. doi: 10.1371/journal.pone.0249792, PMID: 33891614 PMC8064590

[ref14] Hofmann V. Stokburger-Sauer N. E. Wanisch A. Hebborn H. (2021). Masked smiles matter - employee verbal expertise and emotion display during COVID-19. Serv. Ind. J. 41, 107–137. doi: 10.1080/02642069.2021.1873296

[ref15] Kastendieck T. Zillmer S. Hess U. (2022). (un) mask yourself! Effects of face masks on facial mimicry and emotion perception during the COVID-19 pandemic. Cognition Emotion 36, 59–69. doi: 10.1080/02699931.2021.1950639, PMID: 34432603

[ref16] Kaulard K. Cunningham D. W. Bülthoff H. H. Wallraven C. (2012). The MPI facial expression database - a validated database of emotional and conversational facial expressions. PLoS One 7:e32321. doi: 10.1371/journal.pone.0032321, PMID: 22438875 PMC3305299

[ref17] Kim G. Seong S. H. Hong S. S. Choi E. (2022). Impact of face masks and sunglasses on emotion recognition in south Koreans. PLoS One 17:e0263466. doi: 10.1371/journal.pone.0263466, PMID: 35113970 PMC8812856

[ref18] LimeSurvey Project Team Schmitz C. (2012). LimeSurvey: An Open Source survey tool. LimeSurvey Project, Hamburg, Germany. Available at: http://limesurvey.org (Accessed December 11, 2022)

[ref19] Marini M. Ansani A. Paglieri F. Caruana F. Viola M. (2021). The impact of facemasks on emotion recognition, trust attribution and re-identification. Sci. Rep. 11:5577. doi: 10.1038/s41598-021-84806-5, PMID: 33692417 PMC7970937

[ref20] Paiva-Silva A. Pontes M. Aguiar J. de Souza W. (2016). How do we evaluate facial emotion recognition? Psychol. Neurosci. 9, 153–175. doi: 10.1037/pne0000047

[ref21] Pantic M. Patras I. (2006). Dynamics of facial expression: recognition of facial actions and their temporal segments from face profile image sequences. IEEE Trans. Syst. Man Cybern. 36, 433–449. doi: 10.1109/TSMCB.2005.859075, PMID: 16602602

[ref22] Pazhoohi F. Forby L. Kingstone A. (2021). Facial masks affect emotion recognition in the general population and individuals with autistic traits. PLoS One 16:e0257740. doi: 10.1371/journal.pone.0257740, PMID: 34591895 PMC8483373

[ref23] Ruba A. L. Pollak S. D. (2020). Children’s emotion inferences from masked faces: implications for social interactions during COVID-19. PLoS One 15:e0243708. doi: 10.1371/journal.pone.0243708, PMID: 33362251 PMC7757816

[ref24] Sayette M. A. Cohn J. F. Wertz J. M. Perrott M. A. Parrott D. J. (2001). A psychometric evaluation of the facial action coding system for assessing spontaneous expression. J. Nonverbal Behav. 25, 167–185. doi: 10.1023/A:1010671109788

[ref25] Sheldon K. M. Goffredi R. Corcoran M. (2021). The glow still shows: effects of facial masking on perceptions of Duchenne versus social smiles. Perception 50, 720–727. doi: 10.1177/03010066211027052, PMID: 34162278

[ref26] Wegrzyn M. Vogt M. Kireclioglu B. Schneider J. Kissler J. (2017). Mapping the emotional face. How individual face parts contribute to successful emotion recognition. PLoS One 12:e0177239 10.1371/journal.pone.0177239. doi: 10.1371/journal.pone.0177239, PMID: 28493921 PMC5426715

[ref27] Yarbus A. L. (1967). Eye movement and vision. Plenum: New York.

[ref28] Yudiarso A. Liando W. Zhao J. Ni R. Zhao Z. (2020). “Validation of Facial Action Unit for Happy Emotion Detection.” in *Proceedings of the 3rd International Conference on Psychology in Health, Educational, Social, and Organizational Settings*. pp. 360–363.

