# Supplementary material for: The effect of masks on the recognition of facial expressions: A true-to-life study on the perception of basic emotions
Source: Front Psychol. 2022 Dec 22;13:933438. doi: 10.3389/fpsyg.2022.933438 (PMC9815612; doi:10.3389/fpsyg.2022.933438)
Supplement: Supplementary file 1 [file Data_Sheet_1.PDF]

Table S1. Confusion matrix for masked emotional expressions (%).

	(1)	(2)	(3)	(4)	(5)	(6)
(1) Happiness	89.51	1.22	3.09	0.75	1.50	3.75
(2) Fear	1.87	85.02	7.12	1.69	1.34	3.00
(3) Disgust	0.94	21.35	48.31	2.43	24.34	2.62
(4) Sadness	2.81	35.39	26.03	27.34	5.99	2.43
(5) Anger	0.37	17.04	28.84	7.30	42.32	4.12
(6) Neutral	1.31	1.31	0.19	1.87	0.56	94.75

Table S2. Mean percentage of recognizing the displayed emotion without and with a facial mask separately for the female and the male actor.

	**Actress**		**Actor**	
**Emotion**	**No Mask**	**Mask**	**No Mask**	**Mask**
Duchenne	93	81	99	95
Social smile	95	90	90	92
Anger	96	36	51	49
Fear	80	78	93	92
Sadness	79	50	41	4
Disgust	76	52	80	45
Neutral	93	94	94	96

**Table S3.** Certainty. The means how certain the participants were of the perceived emotion separately for the female and the male actor.				
	**Actress**		**Actor**	
**Emotion**	**No Mask**	**Mask**	**No Mask**	**Mask**
Duchenne	6.06	4.82	6.33	5.69
Social smile	6.24	5.22	5.82	5.40
Anger	6.23	3.72	4.39	4.27
Fear	5.24	4.85	5.85	5.29
Sadness	5.21	3.66	5.08	4.03
Disgust	5.42	3.87	5.00	3.82
Neutral	6.04	5.28	6.13	5.60

**Table S4.** Intensity. The mean perceived intensity of the emotion separately for the female and the male actor.				
	**Actress**		**Actor**	
**Emotion**	**No Mask**	**Mask**	**No Mask**	**Mask**
Duchenne	4.48	3.71	4.41	4.73
Social smile	3.82	3.15	3.27	2.97
Anger	4.82	3.29	3.64	4.33
Fear	4.31	4.19	5.42	5.48
Sadness	4.18	3.58	5.07	4.53
Disgust	4.48	3.80	4.78	4.40
Neutral	2.92	2.74	3.03	2.73

**Table S5.** Perceived honesty of happiness separately for the actress and the actor.				
	**Actress**		**Actor**	
**Emotion**	**No Mask**	**Mask**	**No Mask**	**Mask**
Duchenne	2.90	3.18	4.65	4.63
Social smile	2.20	2.87	2.53	2.78

Since OpenFace - naturally - had problems with the analysis of the “mask clips”, we limited analysis only to maskless video clips. In light of the fact that we were meticulous in creating the videos to ensure that the AUs were activated as identically as possible between conditions, it can be assumed that the results from OpenFace provide a satisfying insight into the AUs activated during the different trials. But it should also be mentioned that the analysis with OpenFace can only be an approximation of the "real" activation of the AUs in the course of the emotion displays. Since the presented emotions started and ended neutrally, the values only provide a medial approximation over the course of time.

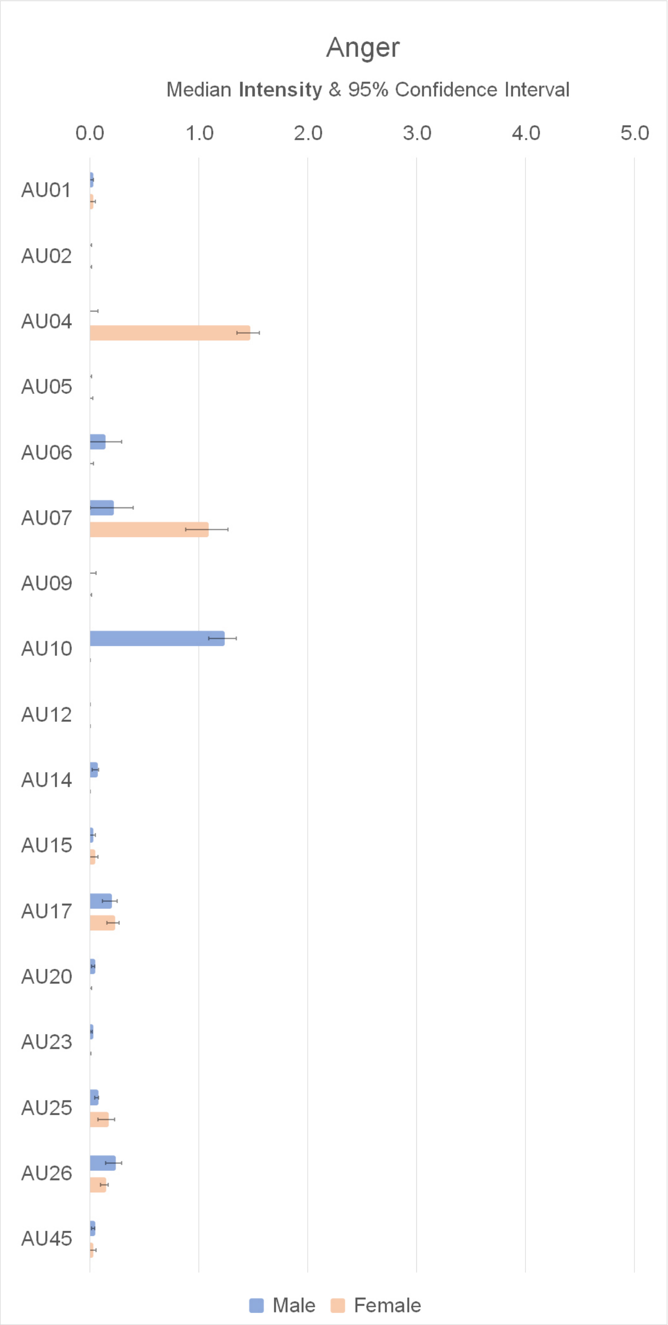

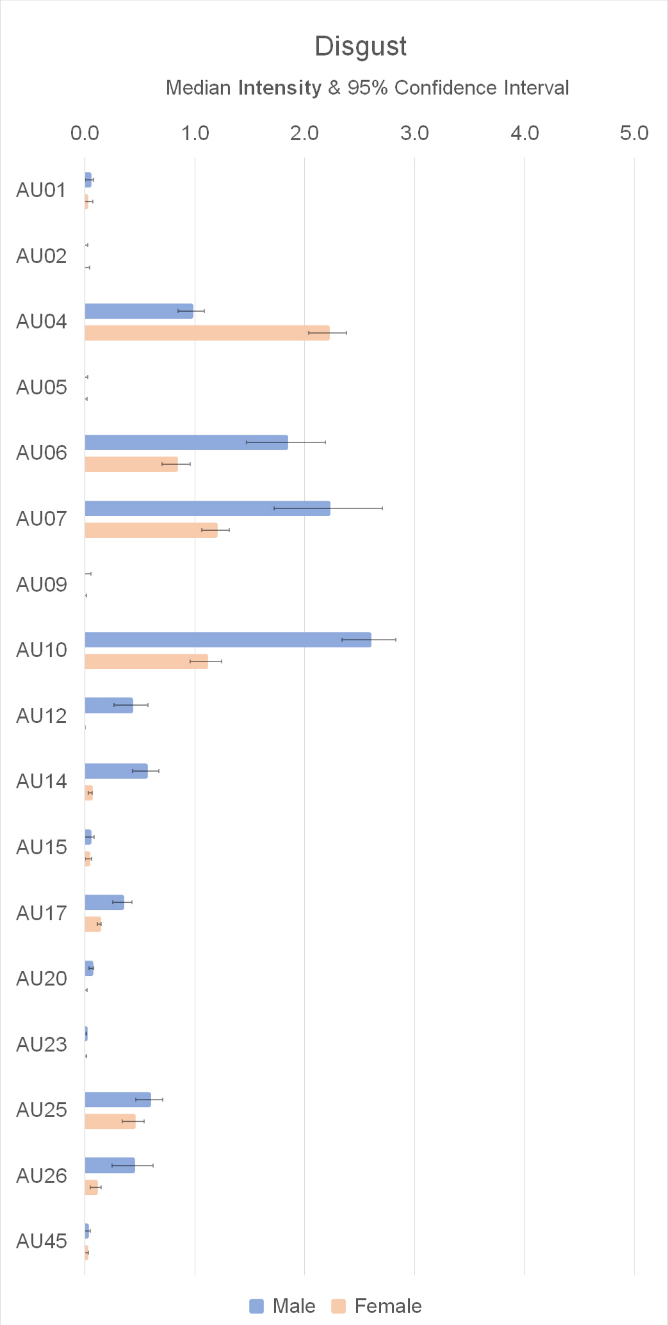

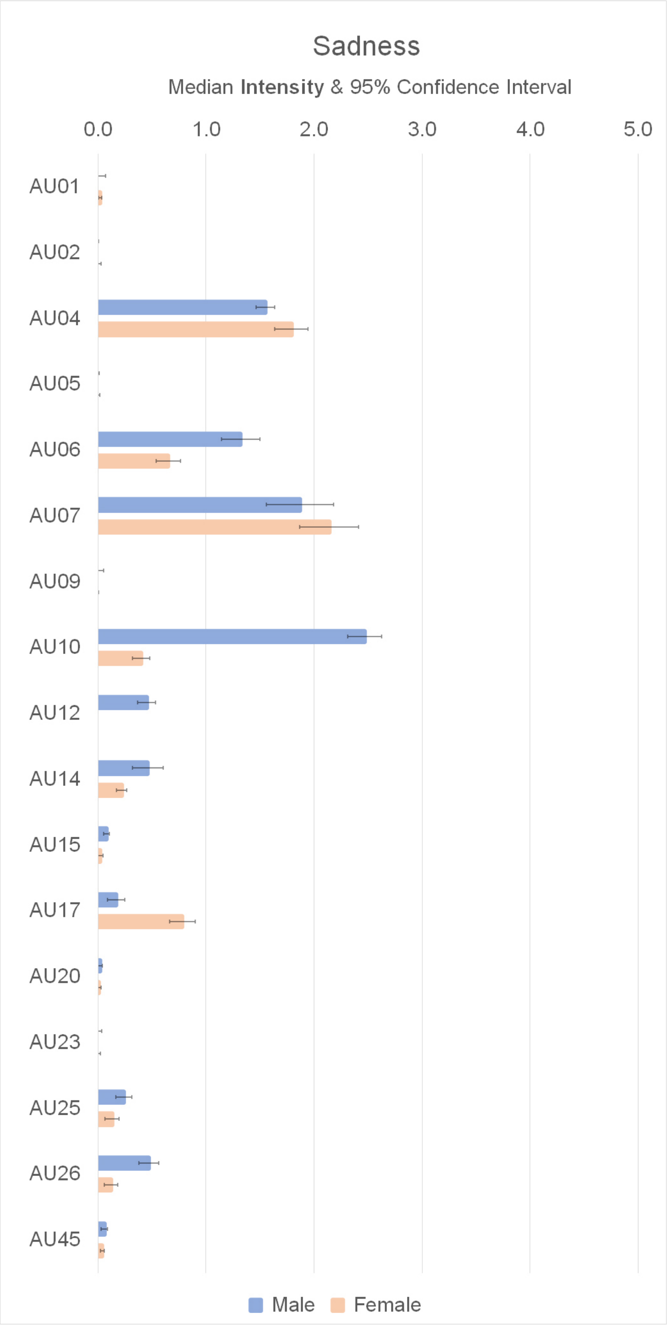

Recording Settings: Mask aligned image / OpenFace Settings: Use dynamic AU models / Face Detector: OpenFace (MTCNN) / Landmark Detector: CE-CLM

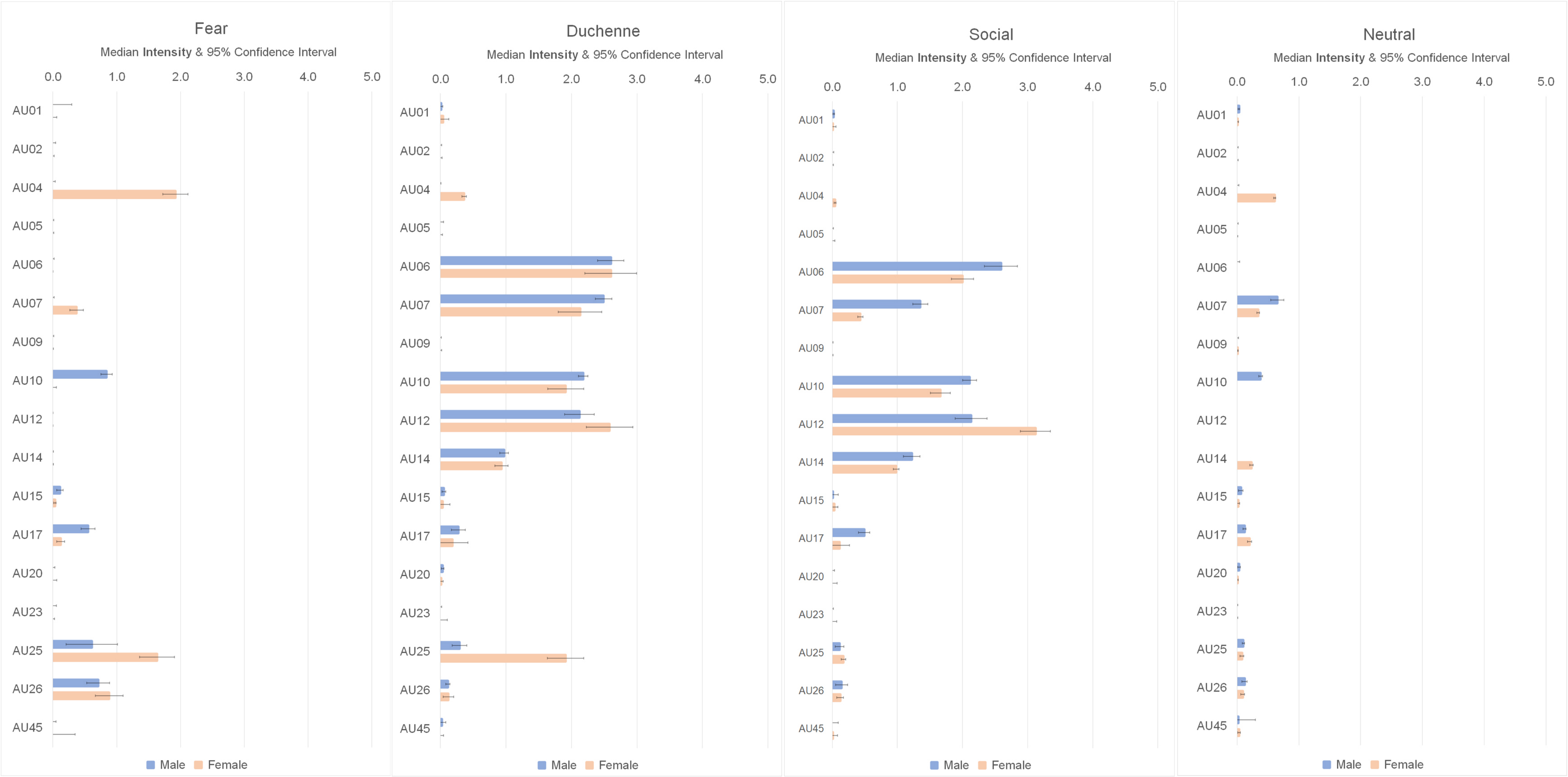

Recording Settings: Mask aligned image / OpenFace Settings: Use dynamic AU models / Face Detector: OpenFace (MTCNN) / Landmark Detector: CE-CLM
